# Efficacy and safety of transcranial direct current stimulation over the left dorsolateral prefrontal cortex in children and adolescents with attention-deficit/hyperactivity disorder: a randomized, triple-blinded, sham-controlled, crossover trial

**DOI:** 10.3389/fpsyt.2023.1217407

**Published:** 2024-01-10

**Authors:** Rachel Silvany Quadros Guimarães, Igor D. Bandeira, Bianca Lima Barretto, Thamires Wanke, Clara Oliveira Carvalho Alves, Thiago Lima Barretto, Chrissie Ferreira de Carvalho, Ingrid Dorea-Bandeira, Arthur Tolentino, Daniel H. Lins-Silva, Pedro H. Lucena, Rita Lucena

**Affiliations:** ^1^Programa de Pós-Graduação em Medicina e Saúde, Faculdade de Medicina da Bahia, Universidade Federal da Bahia, Salvador, Brazil; ^2^Department of Psychiatry and Behavioral Sciences, Stanford University, Stanford, CA, United States; ^3^Faculdade de Medicina da Bahia, Universidade Federal da Bahia, Salvador, Brazil; ^4^Instituto de Psicologia, Universidade Federal da Bahia, Salvador, Brazil; ^5^Departamento de Psicologia, Universidade Federal de Santa Catarina, Florianópolis, Brazil; ^6^Escola Bahiana de Medicina e Saúde Pública, Salvador, Brazil; ^7^Departamento de Neurociências e Saúde Mental, Faculdade de Medicina da Bahia, Universidade Federal da Bahia, Salvador, Brazil

**Keywords:** tDCS, ADHD, non-invasive brain stimulation, dorsolateral prefrontal cortex, executive functions, neuromodulation, randomized controlled trial

## Abstract

**Introduction:**

Although pharmacological treatment for Attention-Deficit/Hyperactivity Disorder (ADHD) has demonstrated efficacy, several individuals persist in experiencing social and academic impairment. Additionally, the occurrence of significant side effects may render the use of psychotropic medications untenable. However, Transcranial Direct Current Stimulation (tDCS), a non-invasive brain stimulation technique, shows promising results in treating ADHD.

**Objectives:**

To investigate the efficacy and safety of tDCS on the performance of children and adolescents with ADHD in neuropsychological tests involving visual attention, visual and verbal working memory, and inhibitory control.

**Methodology:**

This study was a triple-blind, randomized, sham-controlled, crossover clinical trial. The intervention consisted of a daily session of tDCS (2 mA) or sham targeting the left dorsolateral prefrontal cortex (L-DLPFC), for 30 min, on five consecutive days. The primary outcome was change in the Visual Attention Test, Fourth Edition (TAVIS-4) before and after each intervention. Subjects were also evaluated pre and post-tDCS using the Digit Span subtest of the Wechsler Intelligence Scale for Children, Fifth Edition (WISC-V), the Developmental Neuropsychological Assessment, Second Edition (NEPSY-II) Inhibiting Response (IR) subtest, and the Corsi Block-Tapping Task.

**Results:**

Fifteen individuals were included, and no statistically significant difference was observed when comparing the results of the TAVIS-4, the IR of NEPSY-II, and the intragroup Digit Span subtest of WISC-V undertaken before and after the procedure. Adverse events were mainly self-limiting and transient. The participants did not perceive any benefit from tDCS when measured on the Patient Global Impression of Improvement (PGI-I) Scale.

**Conclusion:**

This study did not meet its primary endpoint and found no performance enhancement in any investigated neuropsychological outcomes relating to the intervention group.

## Introduction

Attention-Deficit/Hyperactivity Disorder (ADHD) is a neurodevelopmental disorder that manifests in early childhood and combines inattention, disorganization, and/or hyperactivity-impulsivity. Symptoms appear in at least two different environments, compromising cognitive abilities, such as motivation and executive functions (1).

Given that ADHD has negative repercussions for the daily life of children and adolescents in their social and learning environment and, when left untreated, can lead to disciplinary issues, substance abuse and also is correlated with depression and anxiety, treatment is recommended. The first option usually involves pharmacological treatment with psychotropic stimulants, whether or not with behavioral therapy (2–5). Among the stimulant drugs approved for use by the US Food and Drug Administration are methylphenidate and amphetamines, which work by increasing the amount of dopamine and epinephrine released in the prefrontal cortex (6). However, some of these medications can adversely affect children and adolescents, which can result in problematic therapeutic adherence and the consistency in usage intolerable to the individual (7, 8).

Transcranial Direct Current Stimulation (tDCS) is currently tested for the treatment of several neuropsychiatric disorders (9–13) and is considered safe for use in the pediatric population (14, 15). Evidence shows that tDCS applied to the dorsolateral prefrontal cortex can improve inhibitory control, impulsivity, and decision-making (16).

However, in the adult population, studies using tDCS to treat ADHD have shown contrasting results, with some suggesting improved performance in tests involving attention, memory, and inhibitory control (17, 18). In contrast, others report no difference concerning the sham group (19, 20). Nonetheless, trend-level improvements regarding inhibition and processing speed (though not attention) were found in a recent meta-analysis (21).

In 2014, Bandeira et al. (22) carried out an open-label trial with nine children and adolescents who received anodic tDCS with a current intensity of 2 mA for 30 min over five consecutive days to promote activation of the left dorsolateral prefrontal cortex (L-DLPFC). Increased performance was observed in the Visual Attention Test, 3rd Edition (TAVIS-3) as well as the Inhibiting Response subtest of the Developmental Neuropsychological Assessment, Second Edition (NEPSY-II) (22). These results could have been influenced by tDCS treatment, and the change in performance suggests a greater processing speed and better ability to detect stimuli and switch between activities.

Effects of tDCS in children and adolescents with ADHD were also shown in previous randomized clinical trials (RCT), such as modulation of memory consolidation (23), executive and inhibitory control, cognitive flexibility (24), and reduction in clinical symptoms of inattention and impulsivity (25).

Based on the previous data in the pediatric population, this study aims to reproduce the Bandeira et al. (22) study findings while widening the research scope and ensuring the technique’s safety.

## Methodology

### Study design

This was a triple-blind, randomized, sham-controlled, crossover clinical trial. The study was conducted at the Attention Deficit Hyperactivity Disorder Outpatient Clinic of Professor Edgard Santos University Hospital, Federal University of Bahia in Salvador, Brazil. The protocol for this clinical trial has been published (26), and the trial was registered in the Brazilian Registry of Clinical Trials (ReBEC), which is affiliated with the World Health Organization (WHO).^1^

The study participants were randomly assigned to two groups: the sham group, which did not receive effective stimulation, and the active group, where tDCS was performed. The allocation process was conducted by an individual not involved with the clinical trial using the randomization tool on randomization.com. The resulting allocation list was secured in a sealed envelope and kept by one of the investigators until the first day of stimulation, when the same individual opened it. Only the investigators responsible for performing tDCS had access to the envelopes to ensure that allocation concealment was maintained. After 1 month, the tDCS and sham-tDCS groups were reversed.

### Intervention

The anode was positioned on the left dorsolateral prefrontal cortex (F3 according to the 10–20 system for EEG), and the cathode in the supraorbital region was on the opposite side. The device used was Striat (Ibramed, Amparo-SP, Brazil), approved by the Brazilian National Health Agency (ANVISA). During the stimulation period, the participants engaged in recreational activities involving memory and attention through memory games, such as “Super Lince” and “Genius.” A trained individual performed five sessions (one per day) in the presence of a qualified physician to avoid possible intercurrence. We delivered tDCS treatment for five consecutive days since the same protocol was performed in previous clinical trials (18, 22, 25, 27).

The tDCS procedure involved the application of a direct current of low amplitude (2 mA) for 30 min using two electrodes (5 cm × 7 cm) soaked in saline solution. The current intensity of 1 mA was initially applied for 1 min before it was increased to 2 mA. At 29 min, the device returned to 1 mA at the last minute. Importantly, current strength, duration, and electrode array size had been previously found to be well tolerated (28). To ensure blinding in the sham group, the devices were covered during the sessions, and no participants or their parents had contact with them. To ensure that participants in the sham group were unaware of the sensation of current flow during the procedure, the device was switched on at 1 mA for the first minute, then turned off for 28 min, and reconnected again in the last minute at 1 mA. The families, research subjects, evaluators, and statistician were blind to the allocation groups. To ensure that family members remained blinded, they were asked not to be present in the room during the tDCS sessions. More details about the trial blinding procedures were previously published elsewhere (26). After a month of washout, the groups were switched. Children and adolescents who initially received tDCS moved to the sham-tDCS group and vice versa.

### Participants

The inclusion criteria was comprised of individuals aged 6 to 16 with a diagnosis of ADHD according to the Diagnostic and Statistical Manual of Mental Disorders, Fifth Edition (DSM-5) and confirmed by experienced child neurologists. Furthermore, the child neurologists conducted interviews with the parents of the participants to validate the presence of ADHD symptoms. Additional criteria included right-handedness, literacy, attending school, residency in Salvador-Brazil or within its metropolitan region, not undergoing pharmacological treatment during the intervention week, and EEG without epileptogenic activity. Consent of those responsible for participating in the study was also required before enrollment since all subjects were underage. We excluded individuals with sensory deficits or other neuropsychiatric comorbidities.

### Outcome measures

After selecting the sample according to the aforementioned criteria and child neurology evaluation, children diagnosed with ADHD participated in a neuropsychological assessment to gauge their intellectual level and ability regarding attention, working memory, and inhibitory control. During the screening visit, the investigators assessed the participants and parents with the following instruments:

1. Wechsler Intelligence Scale for Children-WISC-V (29): to estimate IQ, we used the vocabulary subtest, which measures semantic knowledge, and the matrix ratio subtest, which evaluates nonverbal logical reasoning ability.
2. Swanson, Nolan, and Pelham Rating Scale, Fourth Edition (SNAP-IV) (30): The children’s guardians answered this questionnaire, which assessed the diagnostic criteria for ADHD based on DSM-IV.
3. Child Behavior Checklist—CBCL (31, 32): this questionnaire evaluates social competence and behavioral issues in individuals aged 4–18, relying on information from their caregivers or guardians.

After enrolling in the study, the subjects were evaluated before and after the first cycle of tDCS or sham and before and after the second cycle of tDCS or sham. Moreover, neuropsychological tests were used for measuring executive function outcomes:

1. Visual Attention Test, Fourth Edition (TAVIS-4) (33): this assessment is designed for children aged 6–17. The child must press and hold a button on a joystick whenever a target appears on the screen. There are two versions of the test: one for ages 7–11 (target stimulus duration of 6 min) and another for ages 12–17 (target stimulus duration of 10 min). Each task provides scores for various parameters, including reaction time, commission errors, omission errors, and the number of successful hits. “Commission Errors” refer to instances when the child responds when they should not. “Error by omission” represents the lack of response to a target stimulus. The average reaction time, measured in milliseconds, indicates how long it takes for the child to press the button once the stimulus appears on the screen. Task 1 assesses selective attention, where the child needs to press the button when the target stimulus appears. Task 2 involves alternating attention, requiring the child to switch between two types of responses to identify identical geometric shapes of the same color. Task 3 evaluates concentration (sustained attention) through an uninterrupted performance test.
2. Digit Span subtest, as a component of WISC-V (29): measures attention and working memory through auditory tasks involving forward (auditory attention) and backward (working memory) digit recall. The examiner verbally presents a sequence of numbers, and the child’s task is to repeat the numbers in the same order as they were spoken (forward) and then repeat the numbers in reverse order (backward).
3. Corsi Block-Tapping Task (34): aims to evaluate visual working memory. The subject is asked to repeat sequences of touches on various cubes. When reproducing the sequences in the forward order, the test assesses visual attention. On the other hand, reproducing the sequences in the backward order examines the visuospatial sketch of working memory.
4. Inhibiting Response subtest (IR) from NEPSY-II (35, 36): this assessment evaluates the capacity to restrain the desire to engage in a pleasant task, stop an automatic behavior, or switch between stopping and automatic behavior. The examinee is presented with a series of stimuli, such as shapes or arrows, and is required to name the shape or direction or provide an alternative response, depending on the color of the stimulus. Errors may occur when an incorrect answer is given, skipped, or not corrected. Any unanswered items due to time constraints are also considered incorrect errors. Additionally, self-corrected errors are noted when an incorrect answer is subsequently corrected by the examinee. The total number of errors is calculated by summing up uncorrected errors and self-corrected errors for each condition, such as naming (involving the selection of information), inhibition (evaluating the ability to inhibit an automatic response), and switching (assessing the ability to switch attention).

Further details concerning the tests used and their applications can be found in the previously published protocol (26). The primary outcome was the difference in the total TAVIS-4 score between baseline and immediately after the fifth tDCS/sham-tDCS session.

The secondary outcome involved differences between pre-and post-tDCS/sham-tDCS in the Digit span subtest of WISC-V, Corsi Block-Tapping Task, and IR of NEPSY-II.

### Sample size calculation

The sample size was calculated based on data from both our previous pilot study (22) and relevant literature (37). While the pilot study provided preliminary estimates, its limited sample size and larger standard deviation (3.58 pre-tDCS and 2.9 post-tDCS) prompted the inclusion of data from the literature, which offered a more precise estimate of the standard deviation. This standard deviation was used for both groups, as the individuals in the sham group were identical to those in the active group. This choice ensured a more validated approach, as the literature-based standard deviation carries greater weight and reliability. The variable “errors by omission” of the TAVIS-4 was considered the primary outcome. The calculation initially resulted in 11 subjects, with a significance level of 5%, power of 80%, a mean difference between paired groups of 1.2, and a standard deviation of 1.24. Assuming a dropout rate of 25%, the final sample size calculated was 14.

### Statistical analysis

The primary efficacy measure was the change from baseline to treatment day 5 in the TAVIS-4 scores in the tDCS and sham-tDCS groups. Secondary analysis was performed on the change from baseline for the Digit span test, IR, and Corsi Block-Tapping Task. We used random intercepts linear mixed-effects models to analyze continuous outcomes, which can adequately account for associations induced by repeated measurements within participants and automatically handle missing values. Independent models included treatment (2 levels, tDCS and sham-tDCS), time (pre and post-treatment), treatment by time interaction, and participants as random effects. F-statistics assessed the main treatment effects using Satterthwaite’s approximation for degrees of freedom. We estimate the effect size between groups using Cohen’s d and defined cutoff values for small, medium, and large effect sizes as 0.2, 0.5, and 0.8, respectively (38). Significance levels were set at 0.05 and were two-sided. All analyses were conducted using R programming software version 4.2.3, and the ImerTest package was used for linear mixed models (39).

### Ethics approval and consent to participate

This study was approved by the local Institutional Review Board (Medical School of Bahia, Federal University of Bahia, Number: 74002515.9.0000.5577) and followed the ethical principles of the Declaration of Helsinki, 2013. All the parents or guardians of the study participants agreed with the methodology used and signed an informed consent form before participant enrollment.

## Results

**Figure 1:**
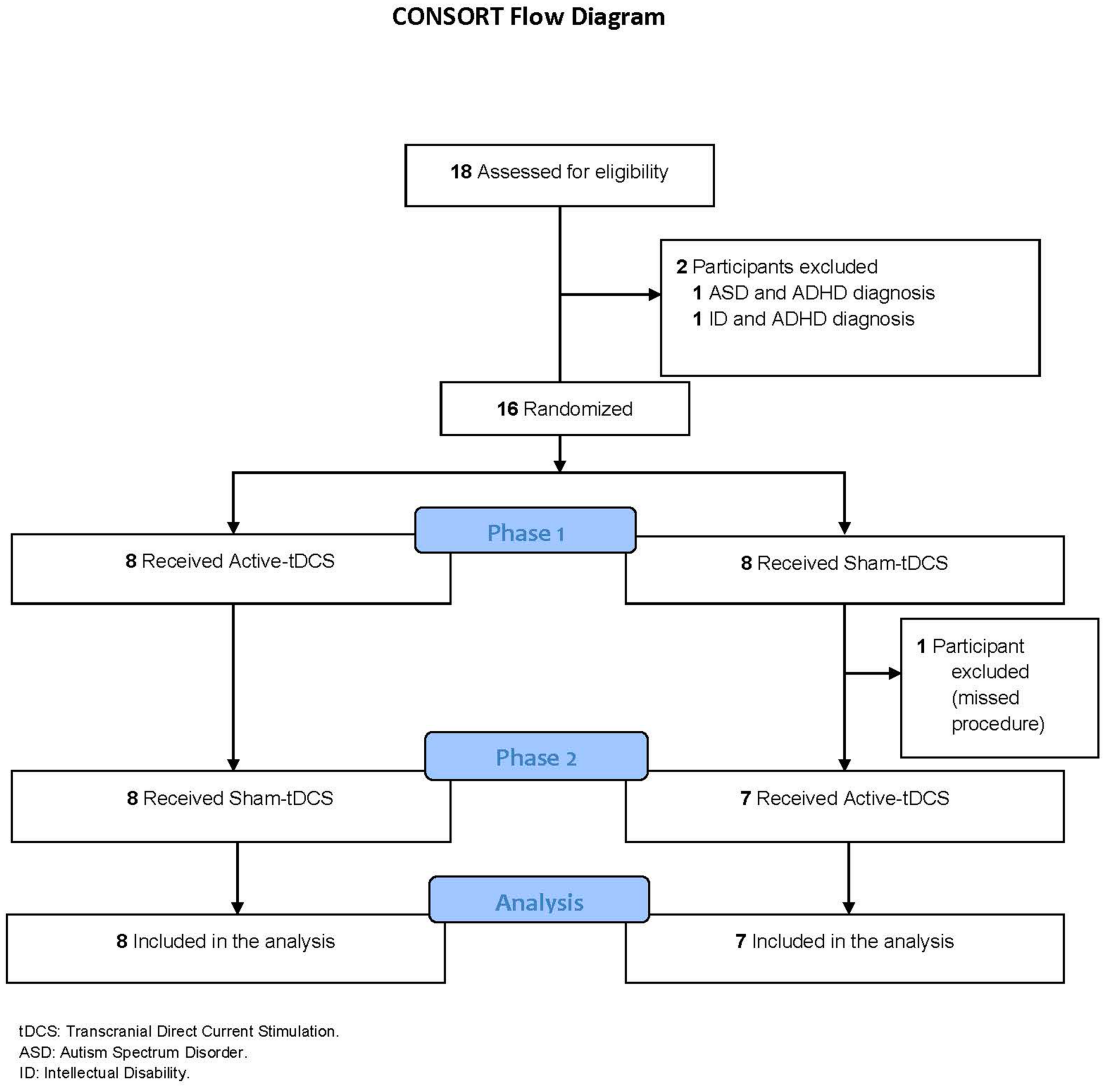
Consort flow diagram.

We assessed 18 children and adolescents for eligibility and randomized 16 into active and sham groups (8 per group). One participant discontinued interventions after finishing five sessions of sham-tDCS, missing the treatment week of active tDCS due to a respiratory infection. Fifteen participants were included in the final analysis (Figure 1). The sociodemographic characteristics of the participants and their guardians can be found in Table 1. Most of the individuals were male (66.67%), black (73%), Latino (100%), had not repeated academic stages (80%), and were born at full term (93.33%). The children were aged from 6 to 15 (Mean: 11 ± 3.1 years), and their IQ ranged from 73 to 105 (Mean: 90.3 ± 10.4). Regarding the level of education, the parent with the best academic level was considered, with two-thirds (66.7%) of them having completed a higher education degree. Regarding the clinical and psychometric profile of the subjects, the mean score on SNAP-IV for attention-related symptoms, hyperactivity/impulsivity, and oppositional defiant disorder were 17.9, 12.6, and 8.6, respectively. According to the cut-off point, attention deficit, hyperactivity/impulsivity, and oppositional defiant disorder were detected in 93.33, 46.7, and 20% of the cases, respectively, having been assessed using SNAP-IV. Detailed baseline psychometric characteristics of individual participants’ data are described in Table 2, including intelligence, executive function, and inhibitory response domains.

**Table 1:** Sociodemographic characteristics of children and adolescents with Attention-Deficit/Hyperactivity Disorder (*n* = 15).

Subject	Sex	Age	Race/Ethnicity	Repeated academic years	Parent/guardian’s level of education
1	M	13	Black/Latino	0	HEC
2	M	9	White/Latino	0	HEC
3	M	10	Black/Latino	0	HEC
4	F	13	White/Latino	0	HEC
5	M	8	White/Latino	0	HEC
6	M	11	Black/Latino	1	HEI
7	F	6	Black/Latino	0	HEC
8	M	8	Black/Latino	0	SC
9	F	15	Black/Latino	3	MC
10	F	7	Black/Latino	0	MC
11	M	11	Black/Latino	0	HEC
12	F	14	Black/Latino	0	HEC
13	M	15	Black/Latino	1	SC
14	M	15	White/Latino	0	HEC
15	M	13	Black/Latino	0	HEC
Mean		11.2 (3.0)			

F, Female; M, Male; EI, Elementary Incomplete; SC, Secondary Complete; HEI, Higher Education incomplete; HEC, Higher Education Complete.

**Table 2:** Psychometric baseline characteristics of children and adolescents with Attention-Deficit/Hyperactivity Disorder (*n* = 15).

Subject	IQ	SNAP-IV: Attention-Deficit	SNAP-IV: hyperactivity	SNAP-IV: ODD	SNAP-IV total	TAVIS-IV: reaction time	TAVIS-IV: errors by omission	TAVIS-IV: actions errors	Digit Span^a^	Inhibition response test^b^	Corsi Block-Tapping Task^c^
1	79	23	8	8	39	2.28	15	11	10	3	7
2	99	18	16	6	40	2.01	12	21	11	6	7
3	100	10	22	16	48	1.92	0	7	11	0	10
4	90	14	3	4	48	1.49	18	18	9	5	9
5	92	19	18	18	55	2.17	19	58	3	27	8
6	84	16	11	7	34	1.94	0	10	6	4	8
7	95	21	23	6	50	2.35	7	4	6	48	4
8	80	20	18	8	46	1.37	12	35	6	4	4
9	73	18	8	12	38	1.59	13	21	8	9	8
10	105	21	24	11	56	2.56	1	111	7	32	3
11	104	20	14	9	43	1.32	19	16	11	0	5
12	97	19	9	15	43	1.61	15	15	6	3	9
13	78	17	8	7	32	1.96	16	53	11	3	8
14	98	15	5	1	21	1.75	9	17	11	3	9
15	81	17	2	1	30	2.19	19	10	15	5	6
Mean (SD)	90.3 (10.3)	17.8 (3.2)	12.6 (7.2)	8.6 (5.0)	41.5 (9.6)	1.90 (0.37)	11.6 (6.8)	27.1 (28.0)	8.7 (3.0)	10.1 (14.0)	7 (2.1)

SNAP-IV, Swanson, Nolan and Pelham-IV; TAVIS-IV, Visual Attention Test-IV.^a^Scores on the direct order of Digit Span test, subtest of the Wechsler Intelligence Scale for Children (WISC-IV).^b^Scores on the Inhibition Response test, subtest of the Neuropsychological Assessment Battery Second Edition (NEPSY II).^c^Scores on the direct order of the Corsi Block-Tapping Task.

### Primary outcome

**Figure 2:**
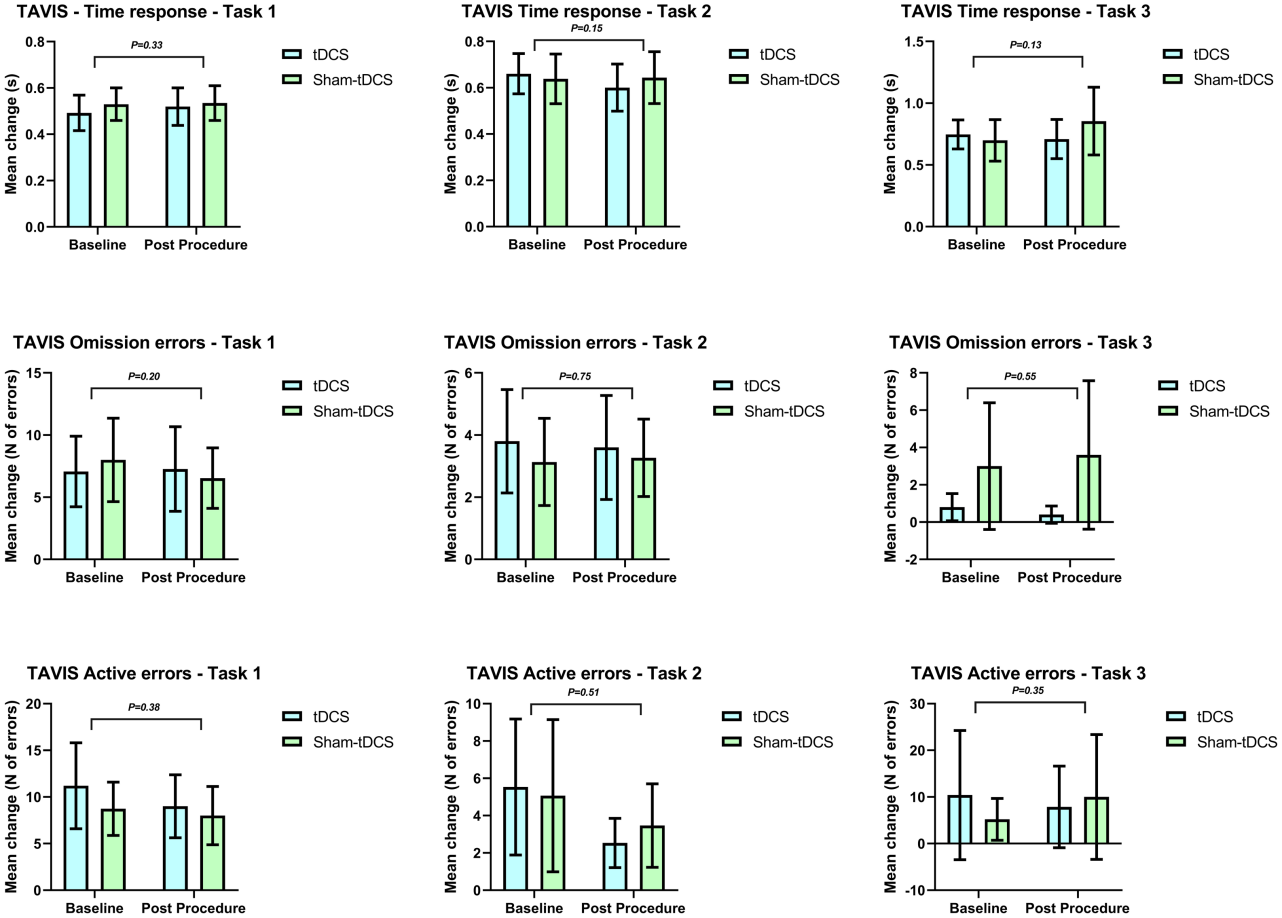
Mean change on TAVIS-4 comparing tDCS and sham-tDCS groups in children and adolescents with Attention-Deficit/Hyperactivity Disorder.

No statistically significant results were found for tDCS treatment. Moreover, when compared to TAVIS-4 between tDCS and sham-tDCS groups, the interaction treatment and time was also not statistically significant regarding reaction time, errors by omission, and commission errors. Mean changes from baseline, confidence intervals, and correspondent effect sizes (Cohen’s d) between tDCS and sham-tDCS groups can be found in Table 3 and Figure 2. Although we found medium effect sizes in some tasks, confidence intervals were wide and encompassed negative values.

**Table 3:** Visual Attention Test (TAVIS-4): estimate of mean change on TAVIS-4 scores after procedures and effect sizes comparing tDCS and sham-tDCS groups in children and adolescents with Attention-Deficit/Hyperactivity Disorder.

Parameters	tDCS (*N* = 15)^a^	Sham-tDCS (*N* = 15)^a^	Analysis			Cohen’s d
			*F*	df	*P-*value^b^	
Reaction time						
Task 1						
Baseline	0.49 (0.13)	0.53 (0.12)				
Change after procedure	0.03 (−0.01 to 0.06)	0.004 (−0.03 to 0.04)	0.97	1, 28	0.33	0.36 (−0.36 to 1.08)
Task 2						
Baseline	0.66 (0.15)	0.63 (0.19)				
Change after procedure	−0.060 (−0.123 to 0.003)	0.005 (−0.053 to 0.069)	2.24	1, 14	0.15	−0.55 (−1.27 to 0.19)
Task 3						
Baseline	0.747 (0.21)	0.698 (0.30)				
Change after procedure	−0.037 (−0.194 to 0.102)	0.155 (−0.034 to 0.366)	2.35	1, 28	0.13	−0.56 (−1.29 to 0.17)
Errors by omission						
Task 1						
Baseline	7.07 (5.12)	8.00 (6.05)				
Change after procedure	0.20 (−1.66 to 2.06)	−1.47 (−3.33 to 0.398)	1.67	1, 28	0.20	0.47 (−0.26 to 1.19)
Task 2						
Baseline	3.80 (3.00)	3.13 (2.53)				
Change after procedure	−0.20 (−1.76 to 1.36)	0.13 (−1.43 to 1.70)	0.09	1, 28	0.75	−0.11 (−0.83 to 0.60)
Task 3						
Baseline	0.80 (1.32)	3.00 (6.13)				
Change after procedure	−0.40 (−3.05 to 2.25)	0.60 (−2.05 to 3.25)	0.35	1, 14	0.55	−0.20 (−0.92 to 0.52)
Commission errors						
Task 1						
Baseline	11.20 (8.31)	8.73 (5.14)				
Change after procedure	−2.20 (−4.77 to 0.37)	−0.73 (−3.31 to 1.84)	0.82	1, 14	0.38	−0.30 (−1.02 to 0.42)
Task 2						
Baseline	5.53 (6.57)	5.07 (7.37)				
Change after procedure	−3.00 (−6.09 to 0.09)	−1.60 (−4.69 to 1.49)	0.44	1, 14	0.51	−0.24 (−0.96 to 0.48)
Task 3						
Baseline	10.40 (24.98)	5.20 (8.11)				
Change after procedure	−2.53 (−16.20 to 11.10)	4.80 (−8.87 to 18.50)	0.92	1, 14	0.35	−0.29 (−1.00 to 0.44)

tDCS, Transcranial Direct Current Stimulation. There was no statistically significant difference between baseline values presented. For Cohen’s d, negative values favors tDCS, whereas positive values favors sham-tDCS.^a^For baseline values, mean and Standard Deviation. For changes after procedures, mean and 95% Confidence Intervals.^b^*P*-values obtained from time by group interaction in Linear mixed effects models.

### Secondary outcome

Outcomes were ascertained by the use of the Digit Span subtest (forward and backward orders), IR (inhibitory control and cognitive flexibility), and Corsi Block-Tapping Task. There was no statistically significant difference regarding therapeutic response between tDCS and sham-tDCS groups (Tables 4–6).

**Table 4:** Digit Span: estimate of mean change on Digit Span scores after procedures and effect sizes comparing tDCS and sham-tDCS groups in children and adolescents with Attention-Deficit/Hyperactivity Disorder.

Parameters	tDCS (*N* = 15)^a^	Sham-tDCS (*N* = 15)^a^	Analysis			Cohen’s d
			*F*	df	*P-*value^b^	
Forward order						
Baseline	8.73 (3.08)	8.33 (2.52)				
Change after procedure	−0.33 (−1.12 to 0.45)	−0.80 (−1.59 to 0.01)	0.74	1, 28	0.39	0.33 (−0.42 to 1.07)
Inverse order						
Baseline	6.73 (1.90)	6.47 (1.72)				
Change after procedure	−0.60 (−1.55 to 0.36)	0.2 (−0.76 to 1.16)	1.46	1, 28	0.23	−0.46 (−1.20 to 0.30)

tDCS, Transcranial Direct Current Stimulation. There was no statistically significant difference between baseline values presented. For Cohen’s d, negative values favors tDCS, whereas positive values favors sham-tDCS.^a^For baseline values, mean and Standard Deviation. For changes after procedures, mean and 95% Confidence Intervals.^b^*P-*values obtained from time by group interaction in Linear mixed effects models.

**Table 5:** Inhibiting Response (IR) subtest: estimate of mean change on IR subtest scores after procedures and effect sizes comparing tDCS and sham-tDCS groups in children and adolescents with Attention-Deficit/Hyperactivity Disorder.

Parameters	tDCS (*N* = 15)^a^	Sham-tDCS (*N* = 15)^a^	Analysis			Cohen’s d
			*F*	df	*P-*value^b^	
Inhibitory control						
Errors						
Baseline	10.13 (14.5)	7.67 (15.7)				
Change after procedure	−0.06 (−5.08 to 4.95)	4.73 (−0.28 to 9.75)	1.91	1, 28	0.17	−0.52 (−1.27 to 0.23)
Reaction time						
Baseline	70.8 (27.9)	74.8 (33.7)				
Change after procedure	−7.45 (−23.60 to 8.66)	1.71 (−14.40 to 17.81)	0.67	1, 28	0.41	−0.31 (−1.05 to 0.44)
Cognitive flexibility						
Errors						
Baseline	14.7 (57.9)	12.1 (X)				
Change after procedure	−1.66 (−7.05 to 3.72)	0.86 (−4.52 to 6.25)	0.46	1, 28	0.50	−0.26 (−1.00 to 0.49)
Reaction time						
Baseline	105.3 (57.9)	102.3 (43.8)				
Change after procedure	−7.7 (−31.2 to 15.6)	−14.8 (−38.2 to 8.6)	0.18	1, 28	0.66	0.16 (−0.58, 0.91)

tDCS, Transcranial Direct Current Stimulation. There was no statistically significant difference between baseline values presented. For Cohen’s d, negative values favors tDCS, whereas positive values favors sham-tDCS.^a^For baseline values, mean and Standard Deviation. For changes after procedures, mean and 95% Confidence Intervals.^b^P-values obtained from time by group interaction in Linear mixed effects models.

**Table 6:** Corsi Block-Tapping Task: estimate of mean change on Corsi Block-Tapping Task scores after procedures and effect sizes comparing tDCS and sham-tDCS groups in children and adolescents with Attention-Deficit/Hyperactivity Disorder.

Parameters	tDCS (*N* = 15)^a^	Sham-tDCS (*N* = 15)^a^	Analysis			Cohen’s d
			*F*	df	*P-*value^b^	
Forward order						
Baseline	7.00 (2.13)	7.73 (2.21)				
Change	0.26 (−0.56 to 1.09)	−0.86 (−1.69 to 0.03)	3.92	1, 28	0.05	0.75 (−0.02 to 1.51)
Inverse order						
Baseline	6.73 (2.31)	6.73 (2.65)				
Change after procedure	−0.46 (−1.55 to 0.62)	0.20 (−0.88 to 1.29)	0.79	1, 28	0.38	−0.34 (−1.08 to 0.41)

tDCS, Transcranial Direct Current Stimulation. There was no statistically significant difference between baseline values presented. For Cohen’s d, negative values favors tDCS, whereas positive values favors sham-tDCS.^a^For baseline values, mean and Standard Deviation. For changes after procedures, mean and 95% Confidence Intervals.^b^*P*-values obtained from time by group interaction in Linear mixed effects models.

Table 7 presents the parents’ subjective perception of therapeutic response after tDCS and sham-tDCS, reporting improvement (mild, moderate, or marked), no change, or worsening (mild) respectively in 2, 8, and 2 of the tDCS group and 5, 8 and 1 of the sham-tDCS group. No significant difference was observed in the perception of improvement between the two groups.

**Table 7:** Perception of therapeutic response after tDCS and sham-tDCS.

Characteristic	tDCS	Sham	*P*
Impression scale of improvement	*n* = 12	*n* = 14	
No difference	2 (16.7%)	5 (35.7%)	0.524
Better	8 (66.6%)	8 (57.2%)	
Worse	2 (16.7%)	1 (7.1%)	
Fisher’s exact test			

### Blinding integrity

Regarding the parents’ perception of the allocation group, it could be seen that, of 26 responses obtained, 16 (61.5%) were in agreement regarding the allocation of the tDCS (9) and sham (7) groups. Of those who disagreed (38.5%), three manifested this after tDCS and seven after sham. Concerning the children’s perception, there were 18 (60%) concordant responses: 10 after tDCS and eight after sham, and 12 (40%) discordant responses: 5 after tDCS and seven after sham (Table 8). The data suggest a low level of agreement, indicating preserved blinding.

**Table 8:** Parents’ and subjects’ perception of allocation group after five sessions of tDCS and sham-tDCS.

	tDCS		Sham-tDCS		Kappa
Parents	tDCS (12)	Sham (12)	tDCS (14)	Sham (14)	0.24
	9	3	7	7	
Subjects	ETCC (15)	Sham (15)	ETCC (15)	Sham (15)	0.20
	10	5	7	8	

### Adverse events

Adverse events were mostly self-limiting and characterized as mild to moderate. Pruritus was identified in 9 (60%) children from the tDCS group and 3 (20%) from the sham-tDCS group. Tingling and burning of greater intensity were reported by 4 (26.7%) and 3 (20%) children, respectively, from the tDCS and sham-tDCS groups (Table 9).

**Table 9:** Adverse events attributed to tDCS and sham-tDCS in children and adolescents with Attention-Deficit/Hyperactivity Disorder.

Adverse Event	tDCS (*n* = 15)		Sham-tDCS (*n* = 15)	
	Mild/moderate	Severe	Mild/moderate	Severe
Headache	5	0	5	0
Itching	4	9	11	3
Tingling	7	4	11	1
Burning	9	3	9	0
Scalp pain	4	0	1	0
Local erythema	13	0	1	0
Irritability	2	0	1	0
Sleepiness	2	1	2	0

## Discussion

Research on brain stimulation has occurred less frequently in the pediatric population than in adults (40), and using cortical neuromodulation techniques in treating neurodevelopmental disorders is a comparatively recent development. However, ADHD remains one of the most studied mental disorders, with a majority of clinical trials involving the anodic stimulation of L-DLPFC (41).

Evidence indicates that variations relating to the stage of the menstrual cycle in which stimulation is performed may be a determining factor for cortical activation, which would then modify tDCS response at an individual level (42). In our study, specific control was not performed according to the menstrual cycle phase during the intervention, which could have affected the results. However, this control would only have been necessary for two of the five females in the study who had menarche.

In a crossover study by Breitling et al. (43), a single 20-min session of 1 mA of anodic tDCS and 1 mA of cathodic tDCS or sham-tDCS was performed, with intervals of at least 1 week between each intervention. The study population consisted of 21 male adolescents diagnosed with ADHD compared to 21 male adolescents who served as healthy controls. Female subjects were not included due to the possibility that menstruation and hormonal factors could affect cortical activation. Although the trial did not include female subjects, the results showed no statistically significant effects in the intervention group.

Westwood et al. conducted a double-blind, randomized, sham-controlled trial testing tDCS in 50 male children and adolescents with ADHD. The active group received anodic stimulation (current of 1 mA, administered for 20 min), associated with cognitive training, over the right inferior frontal cortex. Aligned with our results, this trial also failed to meet its primary endpoint (44). Moreover, in an analysis of a subpopulation of this sample with 23 boys, no significant difference was found in QEEG spectral power during rest and Go/No-Go Task performance. The authors also pointed out the lack of statistically significant findings regarding clinical and cognitive measures in their study (45). These negative findings in the gender-controlled studies previously mentioned suggest that the presence of female participants in our study might not have influenced the observed lack of tDCS effect.

Along with our negative results, many other RCTs have failed to show the superiority of tDCS compared with sham. Schertz et al., performed a randomized, double-blind, sham-controlled pilot study on 25 children, combining cognitive training with the use of anodic tDCS on L-DLPFC three times a week (20 min per session) at an interval of 4 weeks. This study found no difference between the tDCS and sham groups in any of the measures used to assess subjects pre-intervention. This was the case after six sessions, 12 sessions, and 1 month after completing the sessions (46).

Salehinejad et al. performed a sham-controlled trial of tDCS evaluating the executive functions of 22 children with ADHD. The stimulation time in the active group was 15 min, with a current intensity of 1.5 mA. Bilateral anodal left and right DLPFC tDCS did not enhance performance regarding inhibitory control, working memory, and cognitive flexibility (47).

Klomjai et al. performed a pilot randomized sham-controlled crossover study of cathodic tDCS on 11 individuals with ADHD on neurophysiological and behavioral outcomes. The active group received current stimulation of 1.5 mA for 20 min over the L-DLPFC for five consecutive days, 1 month apart. After five active sessions, the study also did not show improvements in attention, only in inhibitory control (27).

Aligned with these previous trials, our study also did not show significant differences for children undergoing tDCS compared to sham-tDCS, contrasting with the positive results of other studies on children and adolescents (16, 22, 24, 25). Due to these studies’ high heterogeneity, it is challenging to define what contributed to the differences in outcomes in the previously published RCT. Possible explanations could be related to the stimulation protocols or the outcomes assessment methods, which can justify the differences between the studies and impair their interpretation, being a confounding factor (21).

Other aspects that may have influenced the results are the tDCS parameters and the simultaneous performance of tasks that require more attention to encourage engagement during the procedure. There still needs to be a consensus in the literature on the influence of simultaneous activities on cortical activation. According to previously published data, performing cognitive tasks to stimulate attention during the application of tDCS is less favorable to the consolidation of neuroplasticity (48). On the other hand, there is data regarding the potential positive use of concomitant tasks during stimulation, such as a previous study testing tDCS for aphasic subjects with simultaneous language training (49).

The current intensity in our trial was 2 mA, higher than 1-1.5 mA used in other studies with children and adolescents (16, 24, 25, 50), which may have influenced the negative results. The behavior of an electrical current in the developing brain can be unpredictable, and the few studies that compared different intensities were carried out using computer models. Although based on estimates regarding anatomical parameters (scalp thickness, subarachnoid space, and skull), these models may not effectively represent what happens in the brain under natural conditions (51).

The allocation order (tDCS or sham) does not seem to have influenced the results. Moreover, the baseline characteristics were similar between subjects since it was a crossover study. Also, despite being a crossover study, there was minimal dropout (only one participant after sham-tDCS).

Adverse events during tDCS were mainly mild and self-limiting, as previously reported (28). The most frequent were pruritus and tingling, in the tDCS and sham-tDCS groups, in accordance with previous studies (25, 50), In addition, there were also reports of a burning sensation and local erythema (mainly in the tDCS group).

Our study assessed responses using the Patient Global Impression of Improvement (PGI-I) Scale. There was no difference in the subjective perception of parents regarding the therapeutic response in the two groups, which reinforces the null effect of tDCS for the parameters used. Furthermore, there was disagreement between the perception of parents and subjects about the allocation group, which shows the preservation of study blinding and the tolerability of tDCS when compared to sham.

Our study has several limitations. First, our small sample size may not have been sufficient to show differences between the groups. However, the sample size calculation was based on differences identified in the literature and our pilot study (22), with a power of 80% and an alpha error of 5%. Secondly, our sample size did not allow for a more robust statistical analysis, controlling for confounding factors. However, some factors minimize this limitation, including our crossover design, which allowed the intervention and control groups to be homogeneous, once they were composed by the same participants. Additionally, we conducted an exploratory subgroup sensitivity analysis with our data based on sex, age, and severity of symptoms; however, no significant differences were observed.

Still, regarding our limitations, we did not select a population with the same ADHD subtype. People diagnosed with ADHD can experience inattention, hyperactivity, or both, linked to a heterogeneous cluster of symptoms and possibly differing regarding functional brain abnormalities. Different stimulation protocols might be needed for each subtype, yet few clinical trials address this issue. The clinical heterogeneity of mental disorders is a challenge in psychiatry research. For instance, a recent study has explored new subgroups of symptom clusters within Major Depressive Disorder, uncovering specific biomarkers (52). However, in ADHD, the current body of data remains insufficient to warrant a study focusing on different subtypes.

## Conclusion

In contrast to previous studies with the same focus, we found no measurable difference in comparison to the sham group in the neuropsychological parameters of visual attention, visual and verbal working memory, and inhibitory control in any of the investigated outcomes involving the application of tDCS for the treatment of pediatric ADHD. In the subjective opinion of the participants, there were no perceptible benefits of tDCS in relation to sham, according to the Patient Global Impression of Improvement (PGI-I) Scale.

## Data availability statement

The raw data supporting the conclusions of this article will be made available by the authors, without undue reservation.

## Ethics statement

The studies involving human participants were reviewed and approved by the Institutional Review Board (Medical School of Bahia, Federal University of Bahia). Written informed consent to participate in this study was provided by the participants’ legal guardian/next of kin.

## Author contributions

RSQG and IDB: conceptualization, data curation, investigation, methodology, project administration, validation, visualization, writing – original draft, writing – review & editing. BLB: investigation, project administration, visualization, writing – original draft. TW, COCA, and TLB: Investigation. CFC: methodology, writing – review & editing. ID-B and DL-S: writing – original draft, writing – review & editing. AT: formal analysis, visualization, writing – review & editing. PHL: formal analysis. RL: conceptualization, methodology, resources, supervision. All authors contributed to the article and approved the submitted version.

## Footnotes

^1^ http://www.ensaiosclinicos.gov.br/rg/RBR-7h5qzf/
